# Health professionals’ perspectives on exercise referral and physical activity promotion in primary care: Findings from a process evaluation of the National Exercise Referral Scheme in Wales

**DOI:** 10.1177/0017896914559785

**Published:** 2014-11-27

**Authors:** Nafees U Din, Graham F Moore, Simon Murphy, Clare Wilkinson, Nefyn H Williams

**Affiliations:** a North Wales Centre for Primary Care Research, College of Health and Behavioural Sciences, Bangor University, Wales, UK; b DECIPHer, Cardiff School of Social Sciences, Cardiff University, Wales, UK

**Keywords:** Exercise referral, health promotion, physical activity, primary health care, process evaluation, Wales

## Abstract

**Background and objectives:**

Referring clinicians’ experiences of exercise referral schemes (ERS) can provide valuable insights into their uptake. However, most qualitative studies focus on patient views only. This paper explores health professionals’ perceptions of their role in promoting physical activity and experiences of a National Exercise Referral Scheme (NERS) in Wales.

**Design:**

Qualitative semi-structured group interviews.

**Setting:**

General practice premises.

**Methods:**

Nine semi-structured group interviews involving 46 health professionals were conducted on general practice premises in six local health board areas. Purposive sampling taking into account area deprivation, practice size and referral rates was employed. Interviews were transcribed verbatim and analysed using the Framework method of thematic analysis.

**Results:**

Health professionals described physical activity promotion as important, although many thought it was outside of their expertise and remit, and less important than other health promotion activities such as smoking cessation. Professionals linked decisions on whether to advise physical activity to patients to their own physical activity levels and to subjective judgements of patient motivation. While some described ERS as a holistic alternative to medication, with potential social benefits, others expressed concerns regarding their limited reach and potential to exacerbate inequalities. Barriers to referral included geographic isolation and uncertainties about patient selection criteria, medico-legal responsibilities and a lack of feedback about patient progress.

**Conclusion:**

Clinicians’ concerns about expertise, priority setting and time constraints should be addressed to enhance physical activity promotion in primary care. Further research is needed to fully understand decision making relating to provision of physical activity advice and use of ERS.

## Introduction

Physical activity reduces the risk of many chronic diseases (Blair et al., 2001), including cardiovascular disease (Lee and Skerrett, 2001), depression and anxiety (Biddle et al., 2000; Stathopoulou et al., 2006), diabetes (Williams, 2007), musculoskeletal conditions (Oida et al., 2003), obesity (Foresight, 2007) and some cancers (Wolin et al., 2007). However, most adults do not achieve recommended levels of physical activity (Chief Medical Officer, 2004). Hence, effective strategies are needed to increase physical activity at the population level and among at-risk groups (Lobelo et al., 2014; NICE, 2006).

Health professionals may play an important role in bringing about lifestyle change among their patients (Chief Medical Officer, 2004; Dugdill et al., 2005; Gidlow et al., 2008; Lawlor et al., 2000; NICE, 2006, 2013; Sowden and Raine, 2008), in part because they come into regular contact with patients at risk from sedentary behaviour (HSCIC, 2012). One increasingly common method for promoting physical activity via primary care is through ‘exercise referral schemes’ (ERS) (Hanson et al., 2013). Although models vary, ERS typically involve referral of patients by a health professional to an exercise programme, consisting of an initial assessment, a tailored programme of exercise and professional supervision.

Systematic reviews of ERS have reported small but significant increases in physical activity in the short term, although long-term effectiveness has been limited (NICE, 2013; Pavey et al., 2011; Williams et al., 2007). There have been concerns about the widespread roll-out of such programmes despite this limited evidence (Pavey et al., 2011; Sowden and Raine, 2008). Hence, attention is needed to understand how the effectiveness of ERS might be improved. Modest effects to date have often been attributed partly to poor uptake and adherence (Hanson et al., 2013; Pavey et al., 2012), and understanding how to better support uptake of and adherence to ERS may serve a significant role in improving outcomes.

In Wales, a National Exercise Referral Scheme (NERS) was established in 2007. Its effectiveness and cost-effectiveness have been evaluated using a pragmatic randomised controlled trial (Murphy et al., 2010, 2012). This found significant improvements in physical activity levels of patients referred with coronary heart disease. For patients referred for mental health problems, depression and anxiety improved, despite a lack of effect on physical activity.

The NERS evaluation included a comprehensive mixed-methods process evaluation, which offered insights into the mechanisms through which adherence and behavioural change were supported (Moore et al., 2013). Some strategies intended to enhance adherence, including motivational interviewing and goal setting (Moore et al., 2013), were not fully delivered. In practice, key components highlighted by professionals and patients as supporting adherence included the patient-only group context, which provided an empathic and supportive environment, and realistic role models for social comparison (Moore et al., 2011b, 2013). The intensive degree of professional contact provided motivational support which was described as enabling patients to build their confidence in using unfamiliar machinery.

While these data provide insights into processes supporting adherence among those taking up referral, attempting to improve uptake requires an in-depth understanding of patients’ journey into ERS, including the referral process. Indeed, patient and professionals’ interviews provided some insights into these issues (Moore et al., 2011b, 2013). Markland and Tobin (2010) have argued that on entry to ERS, behaviour change is typically externally motivated by the advice of the health professional. However, many NERS patients cited a self-determined decision to become more active, actively seeking referral from health professionals, rather than being advised to enter (Moore et al., 2013). This theme was further supported by data from professionals, who argued that the scheme was often more effective for patients who sought referral (Moore et al., 2011b), while quantitative data indicated that patients who were least active at baseline were less likely to complete NERS (Moore et al., 2013). However, mediational analyses showed that effects on physical activity were explained largely through improvements in autonomous motivation, with patients who entered the scheme the least active typically experiencing the greatest improvements in autonomous motivation (Littlecott et al., 2014; Moore et al., 2011a). Hence, while some patients entered NERS with an already high level of internal motivation, for others, NERS supported a movement from externally motivated change (for reasons such as the advice of the health professional) to more internal motivation.

To date, few studies have attempted to understand the referral process from the perspectives of health professionals. The small number of qualitative studies which have examined clinicians’ experiences of ERS has identified a range of barriers to their use by health professionals. These include concerns about giving advice which is not directly linked to patients’ presenting condition, perceptions about patient motivation and the effectiveness of physical activity promotion schemes such as ERS and conflicts with other clinical commitments (Graham et al., 2005; Persson et al., 2013; Singh, 1997). This paper reports interviews conducted as part of the NERS process evaluation programme. It explores health professionals’ perceptions of their role as promoters of physical activity and perceived barriers and facilitators to referring patients into a national ERS in Wales.

## Methods

### NERS

The planned intervention comprised referral to a local authority facility by health professionals. Sedentary people with at least one health condition (e.g. mild-to-moderate depression/anxiety, diabetes, high blood pressure) were eligible. After an initial health check, and motivational interviewing and goal-setting consultations with National Vocational Qualification Level 3 (NVQ3) qualified exercise professionals, patients were offered a discounted (£1 [British Pound Sterling] per session) 16-week supervised exercise programme comprising primarily of group-based patient-only exercise classes. In practice, some core components such as motivational interviewing and goal setting were not fully delivered. For a full description of the intervention as intended, and as delivered, see Moore et al. (2013).

### Participants

General medical practices in six local health boards in Wales within catchment areas of NERS who had referred at least one patient to the scheme were stratified according to geographic region (north, south-west and south-east), practice size and referral pattern. Practice size was categorised as large or small according to the median (*n* = 6,500) number of registered patients. Referral rate was categorised as high or low according to the median referral rate per 1,000 registered patients which was seven. The referral data were obtained from the trial coordinators in each of the health board catchment areas. On a list of 15, 12 purposively sampled practices agreed to participate and comprised four practices from each region, two large and two small, with high and low referral rates, representing both deprived and non-deprived areas using the Welsh Index of Multiple Deprivation criteria (Welsh Assembly Government, 2008). At the end of nine interviews, no new themes were emerging, suggesting that theoretical saturation had been achieved (Ritchie and Spencer, 1994). Hence, no further interviews were arranged.

### Group interviews

All primary health care clinicians and managers of selected practices were invited to participate in semi-structured group interviews lasting up to 1 hour, on practice premises over lunch. Interviews were conducted during the 12-month period of the trial recruitment. Group rather than individual interviews were used to capture the views of a number of participants and the interaction between them (Kitzinger, 1994, 1995). Written informed consent was obtained at the start of the interview. The interviews were conducted by a moderator (ND – a primary health care researcher) and a co-moderator (GM – a social scientist or NW – an academic general practitioner [GP]) using a topic guide containing open-ended questions about the objectives of interest. The topic guide was developed from a thorough literature search and was modified in an iterative fashion after each interview. This study received ethical approval from the Thames Valley Multi-centre Research Ethics Committee (MREC) as part of the larger overall trial (ISRCTN47680448) (Murphy et al., 2010, 2012).

### Data handling and analysis

Interviews were digitally recorded and downloaded onto Cardiff University’s secure computer network before being erased from the recorders. Recordings were fully transcribed verbatim. The transcripts were anonymised and transferred to the QSR^©^ NVivo 8.0 software for qualitative research (QSR International, 2009), which aided management, coding and indexing of the data. One researcher (ND) read the transcripts and coded the data into emerging categories, themes and sub-themes, which were iteratively modified as the interviews progressed. The key points of coded data were indexed and summarised thematically, retaining the context and semantic in which it was expressed, according to the Framework method for thematically analysing the qualitative data (Gale et al., 2013; Ritchie and Spencer, 1994) and were then mapped onto the typology of physical activity promotion and ERS referral behaviours of the participating health professionals. This method is widely used in health care research and provides a flexible but structured way of performing thematic analysis, with the advantage of helping to systematically reduce the data to categories, themes and sub-themes and at the same time retaining links to raw data (Gale et al., 2013; Ritchie and Spencer, 1994).

Anonymised transcripts together with the coded matrix output from the analysis software were then sent to NW or GM corresponding to the interviews that they co-moderated. After their reflections, disagreements were discussed, resolved and codes amended accordingly. This process helped to improve rigour and reduce researcher bias in data analysis and interpretation (Cohen and Crabtree, 2008). Emerging themes and sub-themes were identified and analysed in detail under these categories, with verbatim quotes selected to represent a range of views.

## Results

A total of 46 practice staff participated in the semi-structured group interviews representing nine practices and included 31 GPs, nine practice nurses (PNs) and six practice managers (PMs). Six practices were categorised as large, and all had low referral rates, with four of these located in deprived areas. Three of the practices were categorised as small with two having high referral rate and two of these situated in deprived areas. Characteristics of surgeries and participants are presented in the Table 1.

**Table 1.** Characteristics of the anonymised general practices and study participants interviewed.

Anonymised general practice code	Practice size (large or small)	Referral rate (high or low)	Socio-economic deprivation	Participant characteristics		Interview duration (minutes)
				Role-Gender	Duration (years) in practice	
S1	Large	Low	Yes	GP1-Male	20	49.29
				GP2-Male	8	
				GP3-Female	4	
				PN-Female	15	
				PM-Female	10	
S2	Large	Low	Yes	GP1-Male	20	31.25
				GP2-Male	30	
				GP3-Female	18	
				GP4-Female	10	
				PN-Female	17	
S3	Large	Low	Yes	GP1-Male	10	47.07
				GP2-Male	8	
				PN1-Female	8	
				PN2-Female	20	
				PM-Female	10	
S4	Large	Low	Yes	GP1-Female	18	30.38
				GP2-Male	12	
				GP3-Male	26	
				GP4-Male	20	
				GP5-Male	20	
				GP6-Female	8	
				PN1-Female	17	
				PN2-Female	20	
				PM-Male	18	
S5	Small	High	No	GP1-Female	8	29.20
				GP2-Female	4	
				PN-Female	8	
S6	Large	Low	No	GP1-Male	30	40.42
				GP2-Female	15	
				GP3-Female	18	
				GP4-Male	2	
				GP5-Male	30	
				PN-Female	12	
				PM-Female	15	
S7	Small	High	Yes	GP1-Male	10	29.10
				GP2-Female	6	
S8	Small	Low	Yes	GP1-Female	15	36.14
				GP2-Male	15	
				PM-Female	12	
S9	Large	Low	No	GP1-Male	15	36.00
				GP2-Male	19	
				GP3-Male	5	
				GP4-Female	8	
				GP5-Male	3	
				PN-Female	15	
				PM-Female	10	

GP: general practitioner; PN: practice nurse; PM: practice manager.

Total general practices = 9; total participants = 31 GPs, 9 PNs and 6 PMs; total recording duration = 329 minutes (5.48 hours).

Analysis was performed under three broad categories that emerged from the data, and these were consistent with the ‘a priori themes’ in our topic guide arising from the literature. These were as follow:

1. Beliefs and attitudes of health professionals to physical activity promotion via primary care;
2. Barriers to referral into the NERS;
3. Facilitators to referral into the NERS.

### Beliefs and attitudes of health professionals related to physical activity promotion via primary care

Most health professionals acknowledged the importance of promoting physical activity in order to improve public health. However, recognition of the value of physical activity did not always lead health professionals to report that they actively promoted it to their patients:

I think to me, everyone should be fit and healthy but everybody cannot be. It depends upon the individual. If some individual wants to, yes I will help. But there are people like for example smokers say ‘Oh, I have been like this and I have been fine; so why should I do it now’, you know. You cannot do anything … (S8-GP1)

The tension between recognising the health benefits of physical activity, and a reluctance to promote it themselves, also appeared to be related to health professionals’ view of their role as ‘facilitators’ rather than ‘parents’, with professionals emphasising the importance of individual responsibility. Health professionals commonly saw their role as giving advice when asked for, rather than ‘coercing’ patients into changing their behaviour:

… there are certain issues where individuals should take the responsibility … and people should not see GPs taking on parental responsibility … (S2-GP1)

Many health professionals cited pressures on their time as reasons for not engaging in physical activity promotion. Competing incentivisation from other services such as smoking cessation was cited as playing a role in prioritisation of such services:

In ten minutes’ consultation, you have to go through whatever they (patients) came up with, medication review and something else and then you haven’t much time for this (PA promotion) … we have to ask for smoking because it is in QOF points but not drinking or exercise … (S8-GP2)

Nevertheless, there was substantial variability in professionals’ reports of offering physical activity advice, with some professionals stating that they did routinely promote physical activity. In part, this was described as being linked to personal experiences with physical activity. Physically active clinicians described opportunistically promoting activity during routine consultations, arguing that their own activity level made them a credible source of advice to patients:

… I think it makes a big difference that most of my patients know that I am extremely involved in sports … If I am telling them, to a certain extent they know, something I am doing myself, I think it does make a difference on their acceptance … (S3-GP2)

By contrast, those who were inactive, overweight or cited unpleasant personal experiences of physical activity sometimes described feeling uncomfortable or hypocritical advising patients to exercise:

… my own experience of exercise is it makes you feel (censored) awful really so I mean you can see (pointing to his big physique) that’s why I am not particularly keen on promoting to my patients … and I suppose it is difficult for me in that situation (apologetic). This is a bit hypocritical … (S3-GP1)

Among professionals who argued that they did provide physical activity advice to patients, many described doing so selectively, targeting advice towards individuals they felt would be motivated to change. Such judgements were sometimes based on patients’ physical appearance, conditions, age and/or gender:

I don’t think I’d pick on the very fat …you’ve got to pick on somebody who you think would be motivated … you’re more likely to get uh, I don’t know, young males who are overweight, perhaps, to be motivated in the sense, um, to exercise … with elderly females with arthritic knees, you’re not going to achieve anything there … (S4-GP3)

There was reasonable agreement that a significant life event or a health crisis such as a myocardial infarction could act as a trigger for patients to change their behaviour, making patients more receptive to behaviour modification advice and allowing professionals to provide advice which was directly relevant to the patients’ presenting condition:

… the people who have got some other problem like hypertension, diabetes or cardiac event, they might be a bit motivated because they are quite distressed … that (PA advice) is more appropriate … (S8-GP1)

Although many felt that physical activity advice, recommendation or prescription was out of their expertise or remit, some stated that they did make an effort to offer brief advice as part of patients’ consultation and sign-post them to specialised services if indicated or requested by the patients:

… not in intricate detail because that (PA advice) is not in our expertise anyway but yes 2 or 3 seconds worth of ‘your blood pressure is high, you are overweight, exercise would be useful, get some more exercise’ … then of course one is going to refer those people to leisure centre … (S1-GP2)

### Barriers to referral into the NERS

For some health professionals, ERS were described as an improper use of health budget in the presence of other competing or more serious health problems. These professionals argued that trying to motivate people to do something they had not done for a long time was a waste of money which could be spent either on other services or reducing environmental barriers to population’s physical activity:

Spending money on motivating people is not justified in the presence of more serious health problems and limitation of resources … (S2-GP2)

This notion was further compounded by the perception that such programmes risked perpetuating inequalities, because patients with most need might face greater barriers to access such schemes:

… we’re almost sending a self-selected group already … those that desperately need it won’t go … (S7-GP2)

However, many health professionals felt that ERS could improve the health of high-risk groups if properly targeted, with steps put in place to ascertain the access and reach of these disadvantaged groups:

… the reason that they are chronically diseased is that they have not been active, drink a lot … because that is really what is going to affect their health and that is what is going to affect public health. Isn’t it? You get people to get exercising (via ERS) … (S1-GP1)

… I think it is (ERS) very useful for the overweight, diabetic, ischaemic heart disease patients … if you actually refer them to the gym and they have been contacted, they feel that you have actually done something for them. They are more likely to take an exercise in this way … I think a lot of patients are eager to exercise; more than we think if you give them the opportunity … (S3-GP2)

Some respondents felt that the involvement of health professionals in the referral process was unnecessary, feeling that they ultimately served as gatekeepers, rationing access to prevent the schemes from becoming overwhelmed, rather than actively promoting the service. This was described by some as exacerbated by the fact that referral criteria were so broad that most of their patients were eligible for referral. Hence, professionals needed to apply additional layers of selectivity. Some suggested that patients should be allowed to self-refer without seeing a health professional:

… why not let the patient self-refer? We are just the gate-keepers and stop it being swamped … (S6-GP5)

Concerns about patients’ safety and responsibility after referral were described as stopping some from offering referral to ERS. These appeared largely to stem from health professionals’ lack of awareness that the liability for referred patients’ safety rested with the NVQ3 qualified exercise professionals assessing patients for eligibility to enter the programme. A certificate of fitness would allow patients entry into the supervised exercise programme. Those clinicians who had either been exposed to the NERS themselves or who had been communicated these details described greater comfort in referring:

Certifying a patient fit to exercise had major medico-legal implications and put us off until the issue was resolved … (S6-GP1)

Engagement with the scheme varied considerably in different areas and at difference stages of the roll-out, irrespective of the local area deprivation. This was described as stemming largely from how the scheme was set up, advertised and communicated to general practices. Some reported a lack of information about patient’s eligibility criteria or the nature of the intervention; long delays by the leisure centres in contacting, assessing and accepting the participants into the scheme; cumbersome paper work; and lack of feedback about patients’ progress as major factors that negatively affected their engagement with the scheme:

… I do not know what happened to the patients once we referred … I just tend not to refer any more … (S9-PN)

Rural location or geographical isolation of practices from the main urban centres and the absence of the facilities offering the scheme in the vicinity of practices were cited as major barriers to referral. Health professionals cited concerns that patients would be either unable or unwilling to travel to neighbouring towns to avail the facilities. This view was noted predominantly among clinicians of practices in socio-economically deprived areas:

… The scheme has not been utilised or requested at all because it is not available at the local pool and people have to travel to another town … (S4-PN2)

Many health professionals’ decision to promote physical activity or refer to NERS was affected by uncertainty about the evidence of effectiveness of physical activity advice for various patient groups compared to other health promotion campaigns such as smoking cessation:

The benefits of exercise are established for heart disease and hypertension but what about other conditions? … We know that smoking cessation programmes work but not sure about physical activity advice or schemes … (S8-GP1)

NERS was being offered on an experimental basis in order to build this evidence base. However, many also cited entering patients into a randomisation process as a barrier to referral. Some stated that they found it challenging to explain the trial to patients. In particular, many reported difficulty, or expressed discomfort, with the notion of explaining to patients that they might be randomised to the control group, with entry to the scheme deferred for 12 months. This was described by some as withholding a service from half of patients, with some arguing that they would not refer until the trial was over and they could offer guaranteed entry into the scheme. In contrast, others advocated the need for randomised controlled trials to generate evidence of whether exercise programmes work or not:

… I think it’s acceptable because you need to have some evidence, eventually … ’cause if you have evidence that says it makes a difference, then you’ve got more chance of getting funding … (S9-GP4)

### Facilitators of referral into the NERS

Health professionals who were involved in physical, sports or leisure activities or who had received positive feedback on ERS through their patients cited using such services in their own practice. These professionals expressed a preference towards promoting lifestyle change rather than just handing out medication and regarded ERS as useful alternatives to prescribing that had added a holistic dimension to patient care:

… from our perspective, it’s added a new dimension to health care – it’s something else we can offer … (S7-GP1)

… people who’ve got low mood, I mean it’s much better to give them exercise than to give them tablets … (S9-GP1)

Assessing patients entering the scheme and designing the exercise regimen according to individual ability, taking the patient through a stepped approach to improvement and increasing intensity/duration according to individual progress were regarded as essential ingredients to the usefulness of such programmes. This tailored nature of the ERS, with classes involving people in a similar situation, was described as a valued approach to motivate participants and reduce the intimidation that referred patients might feel when entering the gym. ERS were considered particularly helpful for people who were socially isolated and might be intimidated by gyms. The peer and social support they received during classes separate from the regular general gym users gave them the feeling of being among like-minded people who were more or less in a similar situation, which in turn gave them confidence and empowered them to modify their behaviour:

… they (referred patients) seemed to be happy by the fact that the exercise programme was geared towards them as an individual, rather than a generic exercise programme … Schemes give patients some degree of focus to their lives and increase their social interaction with other people … and they realise that they are in a group of like-minded people and this thing gets rid of that intimidation … (S3-GP2)

There were contrasting views about the initial subsidised offering of the NERS to eligible patients. Health professionals in general considered it a valuable incentive to patients to get them started at exercising. But many in deprived area practices, especially, were concerned that patients might revert to their previous routine once the subsidised period was over because they would not be able to pay the higher costs of using the facilities:

… you may mean that the gym is very expensive for people. What this scheme does is get people exercising. Once you get doing exercise, you do want to do more … so if you can get people started at that reduced cost even though they have to pay the full price afterwards … (S3-GP2)

## Discussion

Consistent with previous research, despite viewing physical activity as important, many health professionals expressed reluctance to promote physical activity via primary care (Lawlor et al., 2000; Laws et al., 2008). Health professionals cited barriers such as a lack of time in the consultation or a lack of incentivisation (Coulter and Schofield, 1991; Graham et al., 2005; Laws et al., 2008; Swinburn et al., 1997). Competing priorities from other health promotion activities such as smoking cessation, which were seen as being both grounded in stronger evidence and strongly incentivised, were also cited as a barrier to promoting physical activity.

In addition, health professionals described a lack of expertise in providing physical activity advice and perceptions that patients may object to lifestyle change advice alongside discomfort telling patients what to do (Lobelo et al., 2009). While previous studies have shown that the likelihood of delivering physical activity advice is shaped by characteristics such as health professionals’ own physical activity (Lobelo et al., 2009; McKenna et al., 1998), this study also found that health professionals report promoting physical activity selectively, making subjective judgements of patient motivation to change based on characteristics such as physical appearance, age and gender.

In addition to provision of physical activity advice more generally, a number of barriers to referring into a national ERS were described. Some were specific to the intervention, and others reflected more general attitudes towards physical activity promotion described above. Key intervention-specific barriers included a perceived shortage of communication and feedback from scheme coordinators at scheme set-up and during roll-out. This is perhaps consistent with patient interviews, reported elsewhere, in which some described having made their health professional aware of the scheme while requesting referral (Moore et al., 2013). Additional barriers included geographical isolation, paper work, lack of clarity regarding medico-legal responsibilities and a lack of financial incentives. Paradoxically, while some health professionals cited a lack of evidence for the effectiveness of ERS as a barrier to referral, others objected to the randomisation process used for the trial.

At a more general level, some considered interventions such as ERS to be an improper use of resources which could be spent on issues such as removing environmental barriers to intervention or expressed concerns that the reach of intervention risked worsening inequalities, due to higher uptake by the ‘worried-well’. Professionals highlighted an inability of the scheme to provide support to every patient who met the referral criteria and hence using additional more implicit criteria to inform a decision to refer, such as judgements about which conditions might benefit most from ERS or which patients might be most motivated to adhere, which may have confounded concerns about uptake being limited to the worried-well.

Perceived difficulties in systematically identifying which patients to refer may in part have driven tendencies for the scheme to be offered largely to patients who had become aware of the scheme by other means and actively sought a referral from their health professional, rather than being advised that it might benefit them (Moore et al., 2011a, 2011b, 2013). Findings that patients play an active role in initiating referral to ERS rather than being passively advised to enter have been reported in other ERS evaluations (Wormald and Ingle, 2004). Hence, the extent to which health professionals’ advice plays an active role in promoting change in ERS or whether advice forms a part of the patients’ journey through a scheme is questionable (Persson et al., 2013). The necessity of health professionals referring to exercise schemes has been questioned previously (Clark et al., 2012). Indeed, some professionals argued that involvement of health professionals was unnecessary and that self-referral should be allowed.

Findings from other components of the NERS evaluation paint a mixed picture regarding the role of professional advice in promoting adherence to ERS or behavioural change. For example, consistent with an assumption that those patients with higher levels of autonomous motivation will engage better, patients who were least active at baseline were more likely not to complete NERS (Moore et al., 2013). However, mediational analyses indicated that patients who were least active on entry to the scheme experienced the greatest improvements in internal motivation, from a lower starting point (Littlecott et al., 2014). Hence, it appears likely that while many patients had made a self-determined decision to change, simply using health professional as a gatekeeper to the scheme, others entered for more externally motivated reasons (health professional advice), with motivation becoming more internalised during the course of the intervention.

It is important to note that, despite some reservations among health professionals, the NERS trial did successfully achieve its recruitment targets within the anticipated timeframes (Murphy et al., 2012), while the scheme has continued to be delivered throughout Wales in the years following the completion of the trial. Hence, while the data presented here suggest a need to further understand the processes through which health professionals make referral decisions, these referral rates indicate both that pragmatic trials of such interventions are feasible and that NERS is seen as a useful referral option by many health professionals.

### Strengths and limitations

This study was part of a larger nested process evaluation (Moore et al., 2011a, 2011b, 2013) alongside a randomised controlled trial of the NERS in Wales (Murphy et al., 2010, 2012) and hence provides insights into referral into a scheme whose effects are established. We initially aimed to interview clinicians in 12 practices, but after nine interviews, theoretical saturation was achieved, so no further interviews were arranged. Participants were representative of the target population from all geographic areas with a range of practice sizes, strength of health care staff and referral patterns, and had fulfilled the aim of ‘purposive sampling’ (Patton, 1990). While group interviews allowed for the incorporation of views of a range of participants, one limitation of this method was that there was hierarchy among the participants, which perhaps led to over-representation of the views of individuals in a greater position of power (Morgan, 1997) such as principals or senior members of staff. At times, more senior health professionals appeared to contribute the most, with PNs or junior staff speaking less or expressing less disagreement. Data were collected at a time while the scheme was being trialled and are situated in that temporal context; whether referral practices or health professionals’ scepticism about some aspects of the scheme changed once feedback on the effectiveness and cost-effectiveness of NERS (Murphy et al., 2012) and key intervention processes (Moore et al., 2011a, 2011b, 2013) was provided could not be captured.

## Conclusion and implications for practice and policy

If physical activity promotion is to be incorporated into routine primary care consultations, clinicians’ concerns regarding skills, priority setting and time constraints need to be considered. Training in physical activity advice-giving as part of continuing professional development might be one way to accomplish this. This could include guidance for professionals on identifying opportunities to provide physical activity or referral to ERS, helping them to avoid reliance upon subjective judgements of patient motivation based on characteristics such as gender, age and physical appearance. Overcoming ideological objections to ‘paternalistic’ aspects of health promotion and encouraging physical activity promotion by health professionals who are not active themselves remain significant challenges. Referral criteria for ERS which are so broad as to make almost all patients eligible are likely to make the process of identifying which patients to refer more challenging for professionals. Hence, ERS should consider stricter targeting of the referral process by defining strict referent criteria as recommended in the National Institute for Health and Care Excellence (NICE) 2014 guidance. Robust mechanisms of communication and feedback on patient progress may be helpful in ensuring continued use of ERS.
